# Personalized Immunotherapy Achieves Complete Response in Metastatic Adenoid Cystic Carcinoma Despite Lack of Conventional Biomarkers

**DOI:** 10.3390/curroncol31100434

**Published:** 2024-09-29

**Authors:** Ünal Metin Tokat, Ashkan Adibi, Esranur Aydın, Eylül Özgü, Şevval Nur Bilgiç, Onur Tutar, Merve Özbek Doğançay, İrem Demiray, Mutlu Demiray

**Affiliations:** 1 Medicana Health Group, Precision Oncology Center, 34750 Istanbul, Türkiye; ashkanadibi.po@gmail.com (A.A.); aydin.esranurr@gmail.com (E.A.); molgenonco@gmail.com (E.Ö.); sevvalnurbilgic95@gmail.com (Ş.N.B.); 2 Division of Cancer Genetics, Department of Basic Oncology, Institute of Oncology, Istanbul University, 34093 Istanbul, Türkiye; 3 Department of Internal Medicine, Cerrahpasa Faculty of Medicine, Istanbul University, 34098 Istanbul, Türkiye; onurcpasa@gmail.com; 4 Yedikule Chest Diseases and Thoracic Surgery Education and Research Hospital, 34020 Istanbul, Türkiye; 5 Department of Molecular Biology and Genetics, Koc University, 34450 Istanbul, Türkiye

**Keywords:** personalized immunotherapy, precision oncology, immune checkpoint inhibitor (ICI), integrative therapies, adenoid cystic carcinoma (ACC), frameshift mutation, splice site mutation

## Abstract

There is currently no effective treatment strategy for recurrent/metastatic adenoid cystic carcinoma (R/M ACC). Furthermore, recent single-agent and combination immunotherapy trials have failed in unselected ACC cohorts, unlike non-ACC salivary gland cancers. Genomic profiling revealed no actionable targets but *NOTCH1* and *KDM6A* frameshift and *CTCF* splice site mutations (no *MYB/L* fusion) with a low tumor mutational burden (TMB), microsatellite stable (MSS) and negative programmed death ligand 1 (PD-L1) were observed. We recommended an anti-programmed cell death protein 1 (anti-PD-1) plus anti-Cytotoxic T-lymphocyte-associated protein 4 (anti-CTLA-4) combination based on TMB 2-fold greater-than-median TMB in ACC, tumor harboring multiple immunogenic frameshift or splice site mutations, and PD-L1 negativity. Accordingly, we achieved a complete response in a radiotherapy (RT) and chemotherapy (CT)-refractory patient with locally recurrent lacrimal gland (LG) ACC and lung metastasis following personalized immunotherapy in combination with integrative therapeutics. Therefore, it is crucial to assess not only conventional immune biomarkers but also patient-specific parameters, especially in “immune-cold” cancer types.

## 1. Introduction

Adenoid cystic carcinoma is a rare type of cancer that typically originates from secretory glands, constituting 1.5–2% of all head and neck cancers [1]. The major salivary glands account for the majority of cases, followed by minor salivary glands [1,2]. Lacrimal glands are structurally similar to the salivary glands, but LG ACCs are much rarer in frequency and much poorer in prognosis [3,4]. Still, ACCs in both anatomical regions share many common properties, including high rates of perineural invasion and convergent genomic profiles [4]. ACC is a biphasic tumor composed of myoepithelial and epithelial cellular components and is divided into three histological patterns: cribriform and tubular (mostly low grade 1/2), and solid (generally grade 3), where the loss of myoepithelial cells is often associated with aggressive solid histology [5]. ACC usually exhibits high rates of locoregional recurrence or distant metastasis, warranting long-term surveillance. The initial treatment plan usually includes surgery and/or adjuvant radiotherapy (based on the surgical margin), as no effective chemotherapy regimen exists. R/M ACC patients are usually incurable, and the systemic therapies are palliative in nature. Therefore, molecularly guided therapies are urgently needed.

The response to immune checkpoint inhibitors (ICIs) has been initially described and approved in unresectable or metastatic tumors (tissue/site agnostic) with TMB higher than 10 muts/Mb [6], and microsatellite instability-high (MSI-H) solid tumors [7]. Although previous studies reported a low TMB in the ACC [5,8], diverse single or dual immunotherapy approaches have been investigated in these patients. The objective response rates (ORR—with or without RT), however, have been scarce in unselected ACC cohorts [9]. Recently, TMB has been subject to debate due to cancer type-dependent variations. Accordingly, multiple new parameters potentially affecting and/or predicting the benefits of immuno-oncology drugs have emerged, including but not limited to cancer type-dependent neoantigenic repertoire [10], clonality/subclonality of the alterations [11], mutational signatures [12,13], type of alterations [14], and functions of the mutated genes and their involvement in immune processes [15]. The efficacy of ICIs may also be improved when combined with CT, targeted therapies, or agents that modify the tumor microenvironment (TME). Combined inhibition of non-redundant PD-1 and CTLA-4 immune checkpoints, especially in tumors with baseline-negative PD-L1 status, increases infiltration and expansion of the activated (not exhausted) effector T cells in the tumor periphery and triggers unique cellular responses compared with monotherapy [16,17]. Likewise, high-dose intravenous vitamin C (IVC) is able to facilitate immune cell infiltration in the TME, thereby augmenting the activity of the ICIs in a T cell-dependent manner with high tolerability and minimal toxicity [18,19,20,21]. Curcumin could suppress immune-related oncogenic pathways such as nuclear factor kappa B (NF-κB), and function as an adjuvant to boost immune response and immunotherapy efficacy [22]. Consequently, the response rates could be improved by a holistic approach encompassing the consideration of not only TMB, MSI, or PD-L1, but also these emerging markers as well as rational drug combinations.

In short, it is critical to perform in-depth characterization of each tumor to offer new treatment modalities to improve patient outcomes and/or quality of life. Here, we achieved a durable and complete tumor regression in an R/M ACC patient through dual immunotherapy and an IVC/bioavailable oral curcumin (BOC) combination despite negative immunotherapy biomarkers. This highlights the importance of considering not only conventional markers but also patient-specific factors. Altogether, personalized immunotherapy could maximize the likelihood of treatment success.

## 2. Case Presentation

### 2.1. Diagnosis and Pathology

**Figure 1:**
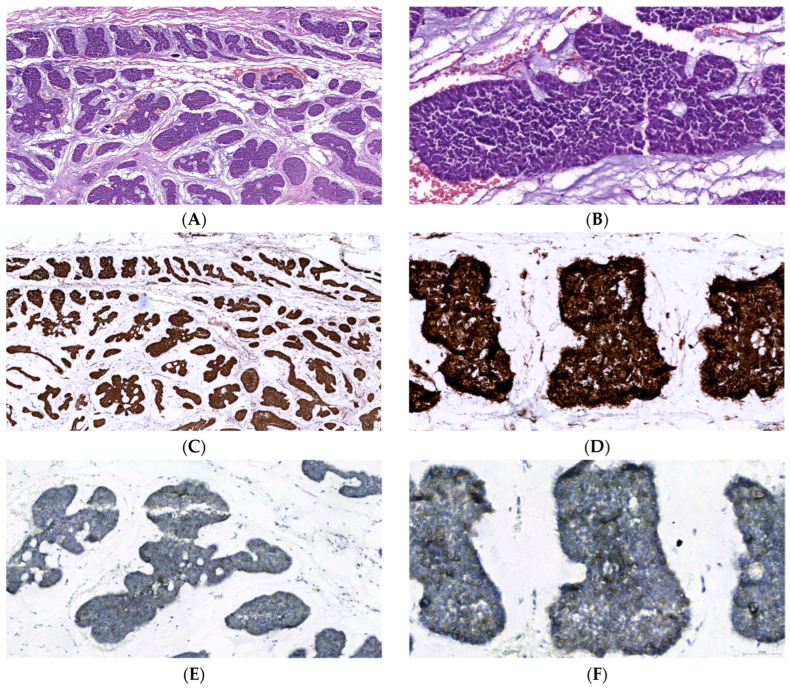
The photomicrograph shows an adenoid cystic carcinoma specimen. There are solid and focal cribriform nests of basaloid cells (Hematoxylin–eosin staining, (**A**)—×100, (**B**)—×400). The immunohistochemistry shows a positive NF-κB p65 p-S536 staining (ab86299 at 1/200 from 0.2 mg/mL stock—final concentration: 1 µg/mL, detected by DAB, (**C**)—×40 and (**D**)—×400). The IHC shows weak-to-moderate focal positive c-Kit (CD117) staining ((**E**)—×200 and (**F**)—×400).

In 2012, a 39-year-old Caucasian female with no family history of cancer or inherited diseases was admitted to the hospital with a swelling in her left eye and was diagnosed with left lacrimal gland ACC (solid variant/type). The patient underwent a lateral orbitotomy with a positive surgical margin. The pathological staging of the tumor was T3N0M0 (stage III—4 × 3 × 1.5 cm). Macroscopically, the solid area was found to continue at the margins of the specimen. Microscopically, the cysts showed an invasive malignant tumor in a stroma with fibrosis and occasional myxoid changes. The tumor was composed of solid and focal cribriform nests of basaloid cells and showed infiltration into partially preserved normal acinar structures and perineural areas (Figure 1). Immunohistochemical (IHC) staining revealed that p63 and calponin were negative in tumor cells, while ESA (EPCAM) and c-Kit (CD117) were diffusely but patchy positive. Tumor cells were stained diffusely positive for phospho-NF-κB p65 (S536) and weak-to-moderate positive for c-Kit in a separate analysis (Figure 1).

### 2.2. Treatment

**Figure 2:**
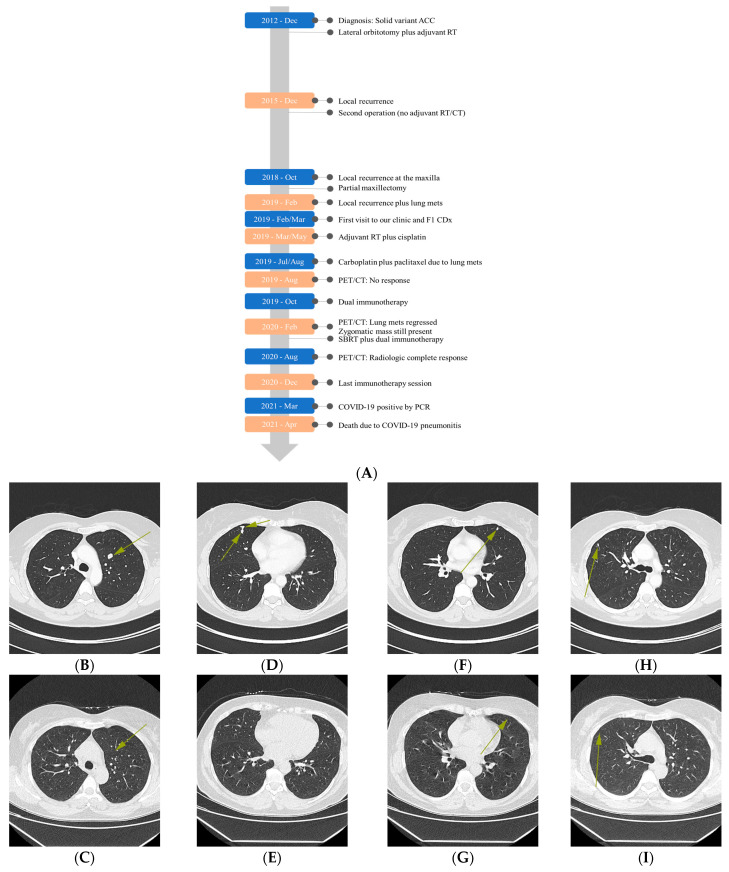
Timeline of disease status and treatment (**A**). CT images showing the presence and absence of lung nodules before and after the treatment (August 2019: (**B**,**D**,**F**,**H**)–August 2020: (**C**,**E**,**G**,**I**)). In A, the spacing between the consecutive boxes (ignoring the box width) on the gray timeline arrow corresponds to the time passed between the events.

In 2012, after receiving a lateral orbitotomy with a positive surgical margin, the patient was treated with adjuvant RT (60 Gray, Gy, in 30 fractions over 6 weeks, 2 Gy per fraction) without CT (timeline—Figure 2). In 2015, a palpable growth at the lower outer quadrant of the eye was observed and was considered a local recurrence by radiologic evaluation. The patient later underwent another operation that removed the orbital contents, bones and adjacent contents with negative surgical margins. No adjuvant RT/CT was planned considering the previous RT and complete resection. In October 2018, a partial maxillectomy was performed due to maxillary recurrence detected during a routine check-up. Due to the positive surgical margin, adjuvant concurrent chemoradiotherapy (CRT-60 Gy plus cisplatin) followed by cisplatin/doxorubicin (75 mg/m^2^ and 60 mg/m^2^) were planned. Although there was a suspected lung metastasis around this time, a mass on the zygomatic bone and ocular cavity as well as multiple metastases in the lung were confirmed in February 2019. The patient was later admitted to our clinic for a second opinion, and we recommended a CGP test to design a personalized treatment plan. Due to post-surgery complications, the patient received adjuvant CRT (60 Gray in 30 fractions over 6 weeks, cisplatin 40 mg/m^2^ weekly) from March 2019 to May 2019. She experienced grade 2 nausea and asthenia. The patient was later offered three-weekly cycles of the combination of carboplatin (AUC 6) plus paclitaxel (200 mg/m^2^) considering the lung metastases, which she refused after receiving two cycles (July–August 2019). Moreover, response evaluation in August 2019 did not show a significant response at primary or metastatic sites.

### 2.3. Comprehensive Genomic Profiling (CGP)-Guided Treatment

**Figure 3:**
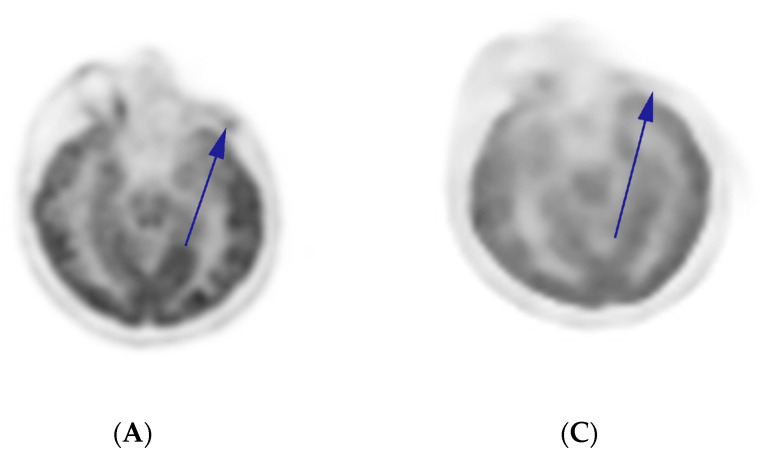

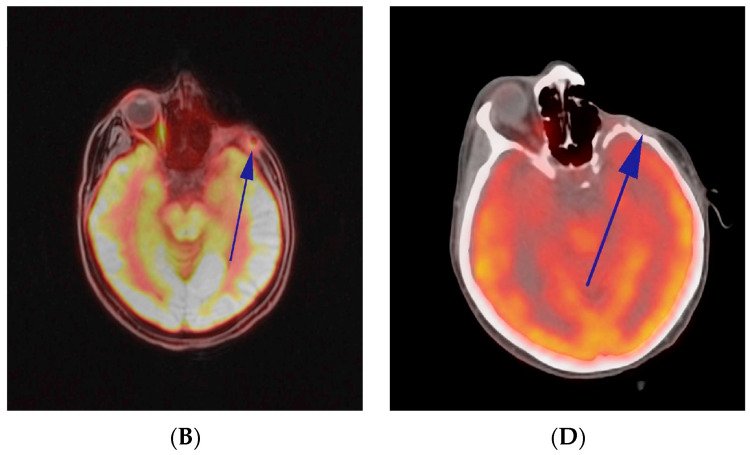
PET/CT images showing marked tumor regression and showing no pathologic FDG uptake following the treatment (pretreatment: (**A**,**B**), posttreatment: (**C**,**D**)). Blue arrows shows that zygomatic mass was eliminated upon treatment.

According to the FoundationOne^®^ CDx (F1 CDx) CGP results (March 2019), the specimen harbors *NOTCH1* D2442fs*35 (VAF: 45.6%), *CTCF* splice site 223 + 1 G > A (48.8%), and *KDM6A* P1107fs*13 (48.2%) alterations with a TMB of 4 muts/Mb, MSS and negative PD-L1 (TPS: 0%-Dako 22C3 pharmDx™). Variants of unknown significance (VUS) include *KDM5A* G1116E (47.2%), *MSH2* A2T (53.9%), *MSH6* A36V (52.1%), *MYC* V280del (29.1%), and *PIK3C2G* Y676H (46.9%). The specimen was negative for any *MYB/MYBL* fusions by the CGP. Consequently, dual immunotherapy (ipilimumab 50 mg in total, nivolumab 400 mg—approved by health authorities) in combination with high-dose intravenous vitamin C (IVC-1.5 g/kg biw on consecutive days; the frequency was later reduced) and BOC (NovoCurmin by Dyna Sci—2 × 2 capsules) were started in October 2019. The BOC was utilized to impair the NF-κB signaling pathway, a strategy which we have successfully used in our previous ACC case based on the same rationale [23]. The IVC, on the other hand, was included to augment the immunotherapy response and induce tumor-selective DNA damage. The control PET/CT scan in February 2020 showed noticeable regression of lung metastases, but the mass on zygomatic bone was still present. We interpreted this as pseudoprogression, but the patient did not consent to a new biopsy. Stereotactic body radiation therapy (SBRT) was planned by an external clinic and she was subsequently treated with SBRT (CyberKnife^®^, ACCURAY, Madison, WI, USA −30 Gy/three fractions). We decided to continue the immunotherapy for 3 more months after also considering the potential abscopal effect and synergy between the immunotherapy and RT. The patient complained of grade 2 asthenia; her thyroid-stimulating hormone (TSH) levels were found to be elevated during this period. She was diagnosed with thyroiditis and later developed hypothyroidism, probably due to the immunotherapy [24]. Accordingly, thyroid hormone replacement therapy was started. Apart from this, the treatment was well tolerated with only mild adverse events (AEs). After 3 months, a complete regression was observed in both lung parenchyma and zygoma. No tumor was observed at 1-year follow-up (Figure 2 and Figure 3). The last immunotherapy treatment was administered in December 2020, and there were no signs of disease progression or serious AEs. The patient tested positive for COVID-19 in March 2021. Overall, we had achieved a radiologic complete response and long-term progression-free survival (PFS, ~17 months) before the patient died from COVID-19 pneumonitis at the end of April 2021.

## 3. Discussion

The current standard of care (SOC) for ACC is surgery followed by adjuvant RT, sometimes in combination with CT. Of the patients receiving RT, more than half will eventually develop local or distant recurrence. Single-agent or combinatorial CT, on the other hand, provided minimal clinical benefit. Therefore, alternative approaches such as targeted agents and immunotherapy should be explored. In this case, we report the successful treatment of a patient with refractory metastatic lacrimal gland adenoid cystic carcinoma. Despite exhibiting a low TMB, MSS, and negative PD-L1 expression (which is typically associated with poor response to immunotherapy), the patient achieved and sustained complete remission following a combined treatment with dual ICIs (nivolumab and ipilimumab), IVC, and BOC. This finding underscores the limitations of relying solely on conventional biomarkers for predicting immunotherapy response and emphasizes the importance of incorporating individualized, patient-specific factors in treatment decisions.

Receptor tyrosine kinase (RTK) inhibitors yielded no-to-low objective responses, although a considerable proportion (~40%) of ACC tumors did harbor alterations in tyrosine kinase genes [8,25]. Therefore, targeted approaches are currently far from being solid treatment options. The frequency of somatic mutations was associated with solid histology [26]. There were frequent alterations in *NOTCH* genes, with the majority seen in *NOTCH1* [8,26]. Unlike *MYB-NFIB* fusions and *TERT* promoter alterations, *NOTCH1* mutations are linked to decreased survival [8] and are more common among tumors with solid histology and liver/bone metastases [8,27]. A separate study reported an ACC subtype characterized by *NOTCH*-activating mutations [unlike loss-of-function (LOF) mutations in HNSCC] and enrichment of solid histology [5]. Furthermore, those with *NOTCH1*-activating mutations had even shorter overall survival (OS) compared with *NOTCH1*-inactivating mutations. Some *NOTCH* alterations could confer sensitivity to gamma secretase inhibitors (GSIs). However, our patient was not expected to benefit from GSI treatment, as nonsense or frameshift mutations removing C-terminal proline, glutamic acid, serine, and threonine (PEST) degron domain stabilize the Notch intracellular domain (NICD) and require ligand-dependent activation or extracellular negative regulatory region (NRR) mutation for complete activation. The mutations in chromatin remodeling genes are also commonly observed in ACC tumors [8,26]. Our patient has alterations in three such genes: *KDM6A*, *KDM5A* and *CTCF*. *NOTCH1* mutations are mutually exclusive with *TERT* alterations, but exhibit a co-occurrence pattern with *KDM6A*, suggesting cooperation between them. Accordingly, *KDM6A* alterations lead to poor survival among ACC patients. CTCF is a transcriptional repressor of c-Myc and could result in MYC upregulation or increased activity when it has an LOF mutation, driving a more aggressive phenotype and disease course [5]. Alterations in PI3K signaling genes (a VUS in *PIK3C2G*) were also typically observed in tumors with solid histology, an aggressive subset of the ACC tumors [26]. In brief, the tumor mutational profile and burden of the patient were compatible with solid-variant ACC, and thus were associated with worse prognosis.

Tumors are not isolated entities but form dynamic interactions with the surrounding stroma, endothelial cells and multiple types of immune cells, enabling them to be treated with alternative therapeutic approaches such as ICIs [28]. To date, a couple of markers have been utilized to predict the response to and benefit of the ICIs [29,30], such as high tissue TMB (tTMB ≥ 10 muts/Mb) [6], MSI-H or dMMR status [31], or high PD-L1 [32]. However, there are many patients without responses to immunotherapy despite the presence of these markers and those with robust responses without these markers. In line with these findings, the threshold for high TMB has been recently questioned, as it is challenging to determine a single, fixed cut-off in a tumor-agnostic manner. TMB failed to estimate the benefit of ICIs in many cancer types [33] and is an inadequate predictor of response to immunotherapy [34]. Previous studies have revealed a low median TMB of 0.3–2 muts/Mb [5,8], and less than 5 and 2% of all ACC cases harbor more than 10 and 20 muts/Mb, respectively [35], excluding >95% of the ACC patients from receiving immunotherapy. Recent studies and meta-analyses also challenged the value of PD-L1 expression as a predictive or prognostic biomarker and revealed that it has limited utility in various cancer types [32,36]. Furthermore, PD-L1 expression is quite uncommon or nonexistent in the ACC specimens [37,38]. There is also no consensus on whether high or low PD-L1 (according to any scoring parameter) or which cut-off for positive or negative staining predicts better clinical outcomes, even across different studies of the same cancer type [39]. On the other hand, PD-L2 was expressed on tumor cells in the majority of primary and metastatic ACC lesions [40] and was associated with cancer cell immune escape/tolerance in HNSCC [41]. Moreover, PD-L2 positivity on combined tumor, stromal, and immune cells in HNSCC significantly predicted clinical response to anti-PD-1 pembrolizumab, independent of PD-L1 [42]. Accordingly, our patient could initially have had a cold tumor microenvironment characterized by low immune infiltration, which would have been transformed into a hot tumor by the anti-CTLA-4 treatment. Subsequently, anti-PD-1 would overcome immune suppression potentially caused by PD-L2 and PD-L1, as both are ligands of PD-1.

A phase 1b KEYNOTE-028 study of pembrolizumab in advanced salivary gland carcinoma (SGC) reported no ORR or durable responses in the ACC [43]. Nivolumab had a low ORR of 8.7% and a non-progressive rate of 33.3% at 6 months in the NISCAHN phase 2 trial in patients with SGC [44]. A phase 2 trial of nivolumab plus ipilimumab in advanced SGC found that the combination had limited efficacy in the ACC [44,45]. However, in-depth analysis of the responding patients (two confirmed and one unconfirmed partial response, PR) revealed that our patient similarly harbored more immunogenic *KDM6A* and *NOTCH1* frameshift/truncation mutations, a potentially higher TMB (4 muts/Mb by F1 CDx—at least 2-fold higher than the median TMB) than the responding patients (25–45 muts by WES) as 306 muts by WES correspond to 8 muts/Mb by F1 CDx [46], and negative PD-L1 status. We recommended dual immunotherapy based on the idea that remodeling the TME by facilitating T cell initiation and trafficking via anti-CTLA-4 could convert the tumor to an immunologically hot state that could be later targeted by anti-PD-1. In summation, the current biomarkers used to guide immunotherapy are insufficient for distinguishing patients who may or may not benefit from the treatment. Therefore, an elaborate examination of all possible contributing factors should be routinely performed during clinical decision-making.

It is critical to follow a patient-centric approach when identifying case-specific immune biomarkers and practice precision immunotherapy. The clonality or subclonality of the alterations present in a tumor have recently been recognized as potential biomarkers. Considering the co-occurrence pattern between the mutations in our patient and their variant allele frequency (~50%), they are likely to be clonal. While clonal neoantigens trigger T cell immunoreactivity and sensitivity to ICI, those of subclonal nature do not [33,47]. Similarly, a meta-analysis concluded that it is not the quantity but the quality of the mutations that determines the efficacy of immunotherapy in a cancer type-dependent manner [48]. Recent studies have also identified several gene alterations and pathways that could predict response or resistance to immune checkpoint blockade (ICB) better than TMB alone [49,50]. For instance, deleterious *NOTCH* alterations causing downregulation of NOTCH signaling in NSCLC or *NOTCH* signaling mutations in colorectal cancer (CRC) were found to be a predictive biomarker of favorable response to ICI [51,52], whereas it elevated NOTCH signaling in small cell lung cancer [53]. In melanoma, *NOTCH1* expression was shown to cause an immunosuppressive tumor microenvironment while its inhibition enhanced immunotherapy efficacy [54]. Conflicting results render *NOTCH1* especially unique, as its role in immunotherapy should be meticulously defined on a per-patient basis, or at least in a cancer type-dependent manner. Similar to *NOTCH1*, *KDM6A* may serve as a favorable or unfavorable marker in metastatic urothelial carcinoma patients with TMB-H or in the general population, respectively [55,56]. Based on our patient’s outcome, we concluded that these mutations might augment immunotherapy efficacy for ACC. Even in mismatch repair (MMR)-proficient tumors, IVC may enhance ICI efficacy by functioning as an adjuvant [18,57], which is currently tested in CRC patients in a pilot study [58]. In multiple preclinical models, vitamin C, together with anti-PD-1 and anti-CTLA-4, induced apparent tumor growth inhibition or regression [18]. IVC also induces oxidative stress and epigenetic reprogramming to cause cancer-selective DNA damage and cell death [59,60,61]. Similarly, curcumin may act as an immunomodulatory agent [22], impair proteasome activity [62], and induce NF-κB inhibitory events to prevent tumor growth and progression in preclinical cancer models [63]. We have previously used it in combination with imatinib and achieved a complete metabolic response in c-Kit and phospho-NF-κB-positive metastatic ACC [23], as both proteins are highly expressed and associated with disease progression in patients with ACC [64,65]. Bortezomib, another proteasome and NF-κB-inhibiting agent, with doxorubicin exhibited high disease control rates in R/M ACC patients in a phase 2 trial [66]. Another important factor that has recently emerged as a predictive marker is the type of alteration. Frameshifts (Fs) and indel mutations carry greater immunogenic potential, as they elevate neoantigen abundance and mutant-binding specificity [67,68,69], partly stemming from a high number of base, and subsequently, amino acid changes that drive changes in protein structure to expand the epitopes’ repertoire [70], unlike SNVs. In a real-world pan-cancer study, tumors with low TMB but Fs alterations had better PFS than those with low TMB without Fs alterations [71]. This finding could pave the way for personalized and potent cancer vaccine development [72,73]. In head and neck cancer, the Fs mutations, including those in *NOTCH1*, were found to be enriched in responders to anti-PD-1/PD-L1 therapies [74]. Pan-cancer splice site mutations may also generate more immunogenic peptides than missense mutations with a positive correlation with high PD-L1 and PD-1 expression and to high T cell immune activity [75]. These genomic parameters could be complemented by immunophenotypic data to better discriminate between immune cold and hot tumors, thus responding to the ICIs [76].

Our study has some shortcomings and limitations. For example, we report a treatment outcome following a dual immunotherapy, IVC, and BOC combination in a single patient, which precludes making generalized assumptions about the efficacy of this treatment in other ACC patients or other cancer types. More studies exploring a personalized approach to treating patients with lacrimal and salivary gland cancers are needed to test the validity of our findings and claims. Second, it may be an oversimplification to treat each and every frameshift and/or splice site alteration as equally immunogenic. This then raises another question about how to precisely assess the immunogenic potential of the genes and these alterations in a personalized medicine setting, although there are tools—albeit experimental ones—that may be utilized for this exact purpose. Finally, we did not have a biopsy taken from the lung nodules to confirm that there was ACC metastasis to the lungs. However, the most frequent site of metastasis in the ACC is the lungs, and radiological monitoring was also compatible with ACC metastasis within the lungs.

Collectively, ACC is an unpredictable and heterogeneous cancer type that exhibits high recurrence risk. Although the majority of the patients could be treated by curative surgery, the current status in the clinic or population-based clinical trials has zero-to-low response rates in (unresectable) R/M ACC, underlying the need for personalized treatment. This necessitates a detailed examination of each alteration and/or potential biomarker in a patient sample to improve treatment outcomes.

## Data Availability

All the data generated for the study are available in this article or from the corresponding author upon request.
